# Nebulized tranexamic acid for recurring hemoptysis in critically ill patients: case series

**DOI:** 10.1186/s12245-020-00304-x

**Published:** 2020-08-20

**Authors:** Fatimah Alabdrabalnabi, Mohammed Alshahrani, Nadia Ismail

**Affiliations:** Imam Abdulrahman Bin Faisal University and King Fahad University hospital, Dammam, Saudi Arabia

**Keywords:** Anti-fibrinolytic agent, TXA, Tranexamic acid, Pulmonary bleeding, Pulmonary hemorrhage, Hemoptysis

## Abstract

**Background:**

Hemoptysis is a clinical condition encountered in the emergency department (ED) and must be managed and investigated urgently to maintain the patient’s hemostasis. The management of hemoptysis depends on treating the underlying cause. Tranexamic acid (TXA) is an anti-fibrinolytic drug used to systemically control bleeding. There are a few studies available that investigate the use of nebulized tranexamic acid for hemoptysis with contradictory results. Our paper demonstrates three cases where patients presented with significant hemoptysis and had significant improvement in symptoms following the administration of nebulized tranexamic acid. The overall need for blood transfusion was reduced.

**Results:**

Three patients presented to the emergency room for evaluation of hemoptysis. All three patients had different underlying pathologies resulting in their hemoptysis and were monitored in the ICU. Initial conventional medical therapies including the correction of coagulopathy and discontinuing offending agents were utilized for treatment. After persistent symptoms, nebulized TXA at a dose of 500 mg three times a day was administered. The patients were all discharged from the hospital with improvement in their symptoms.

**Conclusion:**

Tranexamic acid may be considered in the treatment of hemoptysis regardless of the underlying cause. This may be utilized pending further workup and investigation into the underlying source of the bleeding.

## Introduction

Pulmonary hemorrhage (hemoptysis) results from a variety of causes that can lead to death and/or end with poor prognosis. Emergent interventions are required, but the treatment of hemoptysis depends on treating the underlying cause which may be difficult to uncover. Recently, the use of nebulized tranexamic acid has been utilized as an alternative treatment in case of non-massive hemoptysis. The problem is there is not enough data for the nebulized TXA contrast to the oral, the intravenous, and the topical one which used in many bleeding conditions. Also, the available studies regarding nebulized TXA are few and controversial. Managing hemoptysis is one of the most important conditions in the emergency department. Therefore, the use of nebulized TXA as a treatment option is important [1–3], since it is available, inexpensive, heat-stable, and cost-effective [4].

## Case series

### Case 1

A 31-year-old female presented to the emergency department in King Fahad University hospital in Saudi Arabia with dyspnea. She was found to have an elevated D-dimer in her laboratory studies. A lung perfusion scan was ordered to exclude pulmonary embolism. The scan demonstrated a normal homogeneous pattern of tracer uptake in both lung fields with no obvious areas of peripheral segmental perfusion defects noted in either of lung fields. Also, the CXR was performed and showed a bilateral small atelectatic band as well as a small left pleural effusion in addition to evidence of alveolar hemorrhage. The patient was admitted to the ICU and received 500 mg tablet of TXA orally, antibiotics, 6 units fresh frozen blood, and 1-unit RBCs; after 9 days, she was given 500 mg of TXA intravenously. The patient was diagnosed with SLE and pulmonary hemorrhage and lupus nephritis. The CXR was repeated and it showed improvement. The platelets are improved. The procalcitonin was < 2, which indicated a less likely source of infection and more likely of an inflammatory process likely of her disease. Cultures from her BAL also showed no growth. After 1 week, the patient began to have hemoptysis again. A CXR was done which showed bilateral pleural effusions with bilateral lung bases developing atelectatic changes. Additionally, there were bilateral confluent ill-defined patchy opacities in the mid and lower lung zones with a prominent bilateral central pulmonary vasculature; the computed tomography with contrast shows the pulmonary trunk and main branches with segmental and subsegmental branches appeared well-opacified without evidence of filling defect or aneurysm formation. Lung parenchyma demonstrated multiple bilateral patchy ground-glass opacities and consolidation noted throughout the lung parenchyma predominantly within the lower lobe which was not sparing the pleural surface. It was associated with a tree-in-bud appearance. The patient was started on 500 mg of nebulized TXA every 8 h for 1 day. Five days later, the patient showed improvement as evidenced by stable hemoglobin, no longer requiring more blood products, and was discharged.

### Case 2

Case 2 is a 14-year-old female with known case of G6PD, nephrotic syndrome, and vasculitis with low complement levels. She was admitted to our hospital because of nephrotic syndrome with microcytic hypochromic anemia for investigation with CAP. The patient developed dyspnea in which CTPA was done, and it was negative for pulmonary embolism, but it showed bilateral consolidation with bilateral mild pleural effusion and mild pericardial effusion. She had a sudden episode of headache, and CT head was ordered. During her imaging, she developed status epilepticus that needed multiple doses of lorazepam and phenytoin; then, she was intubated on mechanical ventilation, not on inotropic support. She shifted to MICU. Bronchoscopy was done which shows diffuse alveolar hemorrhage with negative cultures. The patient received 500 g TXA in 5 ml 0.9% NS through the ETT once, then nebulized 500 mg in 5 ml of 0.9% NS every 8 h for 3 days through a nebulizer; the bleeding from ETT is markedly reduced; O_2_ requirements reduce with reduced PEEP; the bleeding stopped whereas there is no positive/negative change on the CXR that is why there is no need to continue TXA. Otherwise, she continued TXA for one more day.

### Case 3

Case 3 is a 66-year-old female with known case of rheumatic heart disease since the age of 13 years complicated with AVR and MVR S/P valvuloplasty and valve replacement in 1978 and 2019; in 1998, she got CAD S/P PCI-atrial fibrillation and was on warfarin; also, she did open heart surgery 40 days back. The patient presented to the ER with shortness of breath and productive cough with hemoptysis for 1 day ago. She has dyspnea at rest with no orthopnea or PND, and no chest pain or bleeding from other sites. She also complained of right upper limb pain for 2 weeks which has worsened over the past few days associated with swelling which started yesterday, no fever, no history of contact with sick patients, and no history of thromboembolism. She was admitted to the ICU and received ciprofloxacin and 500 mg TXA through IVP; the next day, she took 100 mg of nebulized TXA every 8 h for 2 days, and bleeding was reduced over the next few days. The patient was discharged with improvement after 12 days of admission.

## Discussion

Tranexamic acid (TXA) is a 4-amino methyl-cyclohexane-1-carboxylic acid which acts as an anti-fibrinolytic compound that binds to lysine receptors to prevent the conversion of plasminogen to plasmin and inhibits the action of plasmin on fibrin. This prevents fibrinolysis; therefore, hemorrhage control is achieved [1, 2, 5–7]. Evidence shows decreased mortality and complications of bleeding if TXA is administered within 3 h of acute trauma. Also, the use of TXA after anesthesia reduces postoperative bleeding and blood transfusions in coronary artery bypass surgery, otorhinolaryngology, orthopedic surgery, neurosurgery, and liver transplantation. Moreover, a multicenter international clinical trial found that TXA decreases postpartum hemorrhage (> 500 ml after a vaginal delivery or 1000 ml after cesarean delivery). It can be used in treatment within 3 h of delivery as well [5]. Also, it can be used in the treatment of heavy menstrual bleeding, postpartum hemorrhage, and hemophilia [6]. A systemic review published in 2017 summarized a different type of studies that measure the effects of TXA in hemoptysis; one of the studies was done for 2 years prospectively, and it showed a good response of 65% of the patients who were treated with endobronchial TXA [6]. Also, a case series reported 6 subjects with hemoptysis; the bleeding stopped after one dose of a 500 mg/3 ml topical TXA bolus given through a bronchoscope, in two patients. Whereas the other 4 patients were treated with nebulized TXA 500 mg/5 ml 3 to 4 times a day (6–8 h), also, the bleeding stopped after the first dose in all the patients [8]. Moreover, the use of 250–500 mg 2.5 of nebulized TXA every 8–12 h for 4 patients revealed good response within hours to 2 days; one of the patients experienced bronchospasms which were treated [9]. Also, it was used in a patient with stage 4 squamous cell carcinoma of the piriform sinus with airway invasion and the bleeding stopped after 15 min of administering 1000 mg of nebulized TXA [10]. However, a nebulized TXA 500 mg used every 8 h for 48 h in two elderly patients complaining of bronchiectasis and thrombocytopenia results with complete cessation of bleeding [11] which was the same dose we used for our patients and showed the same effectiveness. Komura et al. present a case of massive hemoptysis due to diffuse alveolar hemorrhage from stage 4 lung cancer and anticoagulation for a recent pulmonary embolism, the patient was treated in the emergency department by using nebulized TXA (1000 mg in 20 ml normal saline), the bleeding stopped after 10 min of taking the dose, and she was protected from using an endotracheal intubation [2]. A prospective, double-blinded, placebo-controlled (normal saline), randomized controlled trial (RCT) was established in patients > 18 years with hemoptysis of various etiologies, but they were not critically ill patients such as the cases we report in this series since they exclude the patients who have a massive hemorrhage. These patients were treated with nebulized 500 mg TXA. Patients who take the TXA show resolution of hemoptysis within 5 days; also, they have shorter hospital length of stay, have less need for interventional procedures, and have no side effects. Also, there is a low recurrence rate within 1 year of follow-up. Thereby, the TXA inhalations are a safe and effective treatment of non-massive hemoptysis comparing to placebo. Also, the inhalation form of TXA is better than the systematic since it is faster and more effective. Moreover, the side effects (thrombosis) were more in IV form than in inhalation [1]. A retrospective cross-sectional study of patients < 18 years in the ICU revealed improvement of pulmonary hemorrhage after 1 dose of 250–500 mg every 6–12 h of nebulized TXA. The median of TXA was used in 6 days, and the bleeding stopped after 1 day (the median), with no side effect of the drug and no need for further management [12]. Ng and others reported no response to intravenous TXA, but when he used 250 mg/5 ml of a normal saline nebulizer with a flow rate of 5 l of oxygen/minute over 15 min, the hemoptysis resolved after 2 days of taking the nebulized TXA [3].

## Conclusion

Recent studies have focused on TXA as a primary medical therapy of hemoptysis. Nebulized TXA is an effective and safe option for patients admitted with non-massive hemoptysis. Here, we present three critically ill patients in the ICU with hemoptysis (alveolar hemorrhage) managed using nebulized TXA as part of the management plan. Variations exist in the results of using nebulized TXA. Some patients show no improvement and mild improvement, while others show complete resolution.

## Data Availability

Not applicable
